# Epileptic dyskinetic encephalopathy in KBG syndrome: Expansion of the phenotype

**DOI:** 10.1016/j.ebr.2024.100647

**Published:** 2024-01-18

**Authors:** Eoin P. Donnellan, Kathleen M. Gorman, Amre Shahwan, Nicholas M. Allen

**Affiliations:** a Dept. of Paediatrics, Galway University Hospital, Ireland; b Dept. of Paediatrics, School of Medicine, University of Galway, Ireland; c Dept of Paediatric Neurology and Neurophysiology, Children’s Health Ireland at Temple St., Dublin 1, Ireland; d School of Medicine and Medical Science, University College Dublin, Dublin, Ireland; e School of Medicine, Royal College of Surgeons in Ireland, Ireland

**Keywords:** KBG syndrome, Epileptic dyskinetic encephalopathy, *ANKRD11* gene

## Abstract

KBG syndrome is characterised by developmental delay, dental (macrodontia of upper central incisors), craniofacial and skeletal anomalies. Since the identification of variants in the gene (*ANKRD11*) responsible for KBG syndrome, wider phenotypes are emerging. While there is phenotypic variability within many features of KBG syndrome, epilepsy is not usually markedly severe and movement disorders largely undocumented. Here we describe a novel early onset phenotype of dyskinetic epileptic encephalopathy in a male, who presented during infancy with a florid hyperkinetic movement disorder and developmental regression. Initially he had epileptic spasms and tonic seizures, and EEGs revealed a modified hypsarrhythmia. The epilepsy phenotype evolved to Lennox-Gastaut syndrome with seizures resistant to multiple anti-seizure therapies and the movement disorder evolved to choreoathetosis of limbs and head with oro-lingual dyskinesias. Previous extensive neurometabolic and imaging investigations, including panel-based exome sequencing were unremarkable. Later trio exome sequencing identified a de novo pathogenic heterozygous frameshift deletion of *ANKRD11* (c.6792delC; p.Ala2265Profs*72). Review of the literature did not identify any individuals with such a hyperkinetic movement disorder presentation in combination with early-onset epileptic encephalopathy. This report expands the phenotype of *ANKRD11*-related KBG syndrome to include epileptic dyskinetic encephalopathy.

## Highlights

• *ANKRD11* is a new causative gene linked to epileptic dyskinetic encephalopathy.
• Epileptic dyskinetic encephalopathy can occur in KBG syndrome.
• Epileptic spasms and Lennox-Gastaut Syndrome occur infrequently in KBG syndrome.
• Re-analysis with up-to-date next generation sequencing approaches are important for undiagnosed patients.
• Identified *de novo* pathogenic *ANKRD11* variant (c.6792delC; p.Ala2265Profs*72).

## Introduction

KBG syndrome (OMIM 158050), named according to the initials of the first three patients K, B and G, was first described in 1975. Classic features of KBG syndrome include characteristic dysmorphisms, macrodontia of the upper incisors, short stature, and developmental impairment [1], [2], [3]. The gene identified in KBG syndrome is *ANKRD11* (encoding ankyrin repeat domain 11) and plays an important role in neurogenesis, transcription regulation and epigenetic processes [4]. In addition to the recognised dysmorphism including dental and skeletal anomalies (typically more evident later in childhood), a wider variety of systemic complications have recently been described [3], [5], [6]. While intellectual disability has also been described at the milder end of the spectrum in KBG syndrome (some attending mainstream schools and pursuing employment independently)[7], [8], severe phenotypes are also part of the spectrum, often correlating with loss-of-function variants or disruption in the functional domains of *ANKRD11*.

Seizures occur in KBG syndrome, mostly as part of a developmental encephalopathy [9], [10], [11]. Recently, Buijsse *et al* reported 26/75 patients with epilepsy (original and historical literature combined), most of whom were classified by International League Against Epilepsy (ILAE) criteria, as having generalized epilepsy (15/38 or 39 % patients), focal epilepsy 12/38 (31.6 %), or combined focal and generalized epilepsy (11/38 or 31.6 %)[10]. Distinctive epilepsy syndromes were identified in only few patients. The median age of onset of seizures is approximately 3 to 4 years, with most patients reaching seizure remission. However, approximately one quarter of patients had a drug-resistant epilepsy [10]. While no genotype-phenotype correlation is clearly identified for the presence of epilepsy characteristics in KBG syndrome, those patients with epilepsy had worse developmental outcomes [10].

Here we describe a presentation of KBG syndrome characterised by a severe early-onset developmental and epileptic encephalopathy (DEE) with dyskinesia (or epileptic dyskinetic encephalopathy), accompanied by a further developmental regression and evolution to Lennox-Gastaut syndrome (LGS). Upon reviewing the literature on KBG syndrome, this unusual and severe presentation in KBG syndrome represents an expansion of the clinical phenotype, adding *ANKRD11-*related variants to the other genetic encephalopathies associated with early onset movement disorder and seizures.

## Patient description

A full-term male infant was referred at 4 months of age for “jittery” movements of limbs and poor feeding. Antenatal, perinatal and neonatal course prior to presentation were unremarkable and developmental milestones were appropriate. Neurological and systems examination, apart from mild intermittent peripheral increased tone, were otherwise normal. Head circumference was 41 cm (25th centile). Initial baseline biochemistry and metabolic screen, and cranial ultrasound were normal. At age 5 months he developed episodes of extensor posturing. A CT and MRI brain showed benign increased extra-axial CSF spaces, but no other abnormality.

**Fig. 1a:**
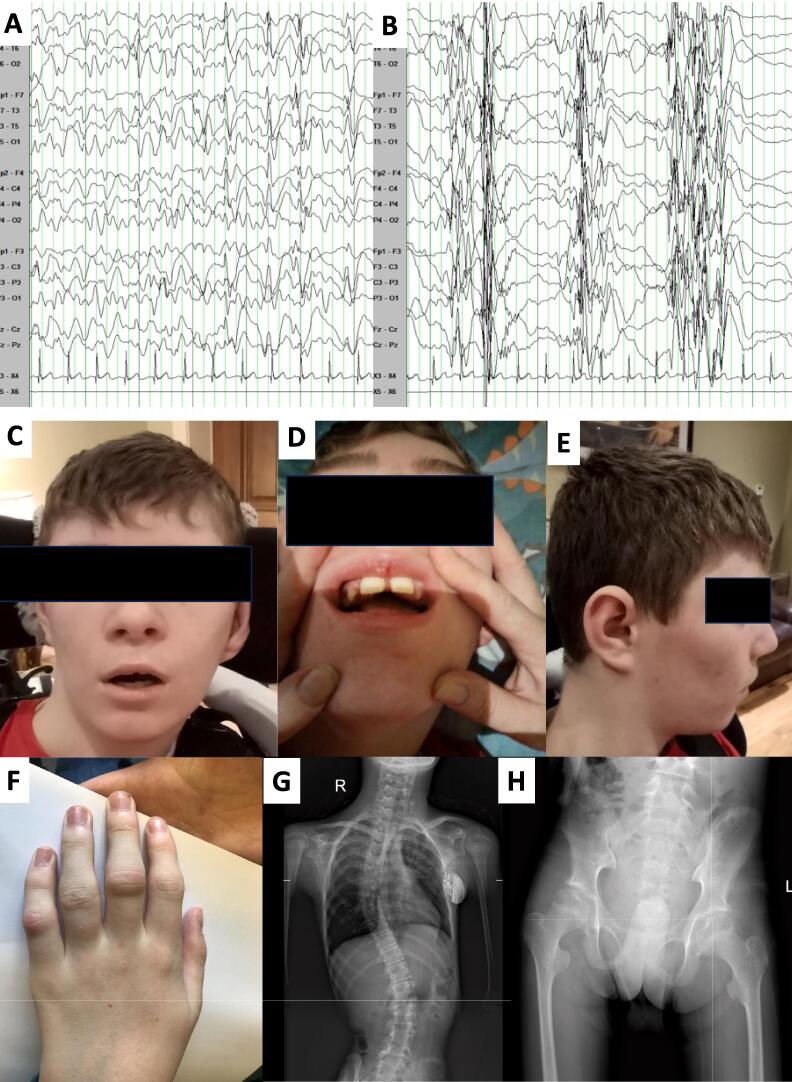
Electroencephalogram (EEG) in awake state showing very slow background and bilateral multifocal slow spike-wave discharges (+/- 1 Hz, <2.5 Hz), (B) in sleep, showing much higher amplitude and impressive bisynchrony with electrodecrements. (C) Triangular facies with anteverted nostrils, bushy eyebrows, long philtrum. (D) Macrodontia of upper central incisors, (E) Brachycephaly. At age 12 years, (F) hand showing clinodactyly of 5th digit, (G) X-ray spine showing convex thoracic scoliosis to the right with lumbar scoliosis to the left (VNS in situ), and generalised osteopenia and (H) narrow underdeveloped pelvis and femora.

The jittery movements persisted, and by nine months he had experienced developmental regression across all domains; no longer able to sit, move on all fours or pull to stand, nor reaching for objects. He stopped smiling socially, could only fix on objects briefly and exhibited nonspecific babbles. He was also noted to have jittery head and hand movements, and paroxysms of trunk stiffening as well as staring. An electroencephalogram (EEG) showed an active epileptic encephalopathy characterised by generalised (bi-synchronous) high amplitude sharp waves, almost continuous at times, with periods of marked background attenuation and electro-decrement during sleep. He had short generalised tonic seizures, as well as generalised epileptic spasms. By the age of 2 years, seizure activity was largely characterised by generalised tonic seizures, atonic seizures and epileptic spasms, refractory to multiple therapies (including corticosteroids, sodium valproate, clobazam, ACTH, vigabatrin, levetiracetam, lamotrigine, topiramate, rufinamide, phenobarbitone, and the ketogenic diet). Accompanying EEG findings were consistent with LGS (Fig. 1**A & B**). The early-onset involuntary jittery movements of head and arms progressed to a mixed hyperkinetic movement disorder. During wakefulness he became restless (with leg kicking, arms waving about), developed stereotypies (headbanging, rocking, teeth grinding, tongue protrusion), gratification, and choreoathetosis involving head, arms, and legs (sometimes ballistic), unrelated to seizure activity, and dissipating in sleep (Video 1).

A vagal nerve stimulator (VNS) was inserted at 5 years of age, which reduced motor seizures and frequency of status epilepticus. Currently, at age 15 years he is treated with multiple ASMs (sodium valproate, phenobarbitone, nitrazepam), with ongoing daily seizures (tonic, tonic-clonic, atypical absences). At age 15 years, the hyperkinetic movement disorder is prominent and includes choreoathetosis of limbs, head, as well as oro-lingual dyskinesia, with episodes of agitation, and discomfort requiring oral clonidine. He is profoundly neurologically impaired (Video 2), non-verbal and non-ambulant. Earlier childhood was also complicated by recurrent otitis media, scoliosis, gastrostomy and precocious puberty.

Throughout the course of illness, genetic testing (karyotype, array CGH, FISH, *MECP2, POLG1*, 127-epilepsy gene panel) and extensive metabolic blood and CSF screens were undertaken (including neurotransmitters), which were all negative. A repeat MRI remained largely unchanged, showing minimal abnormality (widened subarachnoid spaces with slightly enlarged cisterna magna). Trio exome sequence analysis (age 12 years) identified a *de novo* pathogenic heterozygous frameshift deletion of *ANKRD11* (c.6792delC; p.Ala2265Profs*72). Upon variant identification, he was noted to have dysmorphic craniofacial characteristics including large upper central incisors, consistent with the clinical features of KBG syndrome which are often noted in later life (Fig. 1**C-E**).

## Discussion

We report a novel presentation of KBG syndrome of severe developmental epileptic-dyskinetic encephalopathy, characterised by early-onset relentless hyperkinetic movements, epileptic spasms and LGS. Although not an ILAE-recognised term, the term epileptic-dyskinetic encephalopathy is a recently applied umbrella description for a group of genetic disorders occurring in the first year of life [12], usually presenting with intractable involuntary (hyperkinetic) movements (dyskinesia, chorea, athetosis, dystonia or tremor) and co-existing, often severe, drug-resistant epilepsy. Seizures can present with, or without, a co-existing defined early-onset DEE syndrome (e.g. EIEE-BS, IESS) and the dyskinesia can be mild or more overt, and even ballistic in nature. There is often a high rate (up to 82 %) of genetic diagnosis [13] and more commonly associated genes identified for this presentation include for example *ARX, STXBP1, GNAO1, FOXG1, SCN8A, GRIN2B, CDKL5, GABRA2, CYFIP2, EEF1A2, SCN8A, SCN2A, SCN1A, GRIN1, GRIN2A, PIGP, HECW2, TBL1XR1, ALG13, SETD5* [12], [13], [14]. *ANKRD11*, although not previously associated with this phenotype, should now be added to this list of causative genes linked with epileptic-dyskinetic encephalopathy, particularly when seeking to correlate variant identification to clinical presentation, as the dysmorphic and other clinical features of KGB syndrome are not usually present until later childhood. As evidenced in this individual, *ANKRD11* was not included in our initial early-onset epilepsy 127-gene panel approach performed in 2015 [15].

We could not identify any other patients in the literature with such a florid presentation of dyskinetic-epileptic encephalopathy in the setting of KBG syndrome. While tic disorders have been described as part of a wider neurodevelopmental disorders in older children with KBG syndrome [5], [7], [16], other hyperkinetic disorders have not. *ANKRD11* plays significant roles in chromatin remodelling essential for gene regulation, neurogenesis and neuronal specification, and is widely expressed across all brain regions including those relevant to extra-pyramidal movement disorders: basal ganglia, thalamus and cerebellum. Several patients with basal ganglia involvement in *ANKRD11*-related KBG syndrome (one with basal ganglia microangiopathy [17] and two with basal ganglia abnormalities [18]) have been reported but are not described to have a movement disorder. Several other malformations of grey matter development such as heterotopia and cortical dysplasia have also been described [3], [19].

While epilepsy syndromes are uncommon in KBG syndrome, a recent multicentre study combining original and historical patients identified 12/63 (19%) epilepsy-affected KBG patients with an epilepsy syndrome (most were generalised epilepsy syndromes) and with associated developmental and/or epileptic encephalopathy (DEE) [10]. Here we describe a patient with LGS, a phenotype also described in three other patients with KBG syndrome [10], [19]. KBG syndrome-affected patients with epilepsy have a significantly poorer neurodevelopmental outcome compared to those without epilepsy. Our patient had profound neurological impairment with global developmental delay and other complications. There are many factors that contribute to a more severe neurologic phenotype in KBG syndrome. Li *et al,* described a greater degree of ID, with variants affecting the R2D domain (affected in our patient), although this molecular association has not yet been a confirmed association for KBG patients with epilepsy (possibly due to lack of power or small numbers of patients affected to date)[10], [20]. Other small series have shown that males with KBG syndrome are more severely affected than females, although the reasons for this remain unclear [6], [8], [21]. Interestingly the pathogenic *ANKRD11* variant in our patient (c.6792delC; p.Ala2265Profs*72) affects the same amino acid position in a female with KBG syndrome (c.6793delC; p.Ala2265Profs*72)[22] who also had seizures (apparently less severely affected, although the full phenotype was not available upon contacting the authors). Another female was reported with KBG syndrome and ID (IQ of 56) due to an *ANKRD11* duplication (c.6792dupC, p. P2271Pfs*8)[20] at a similar position to the individual described here but also had a much milder phenotype, and no report of epilepsy or movement disorder. It is possible that other genetic factors could account for phenotypic heterogeneity including the interactions of other transcription regulating genes or environmental factors [10]. It is also known that patients with contiguous gene deletions (involving 16q24.3 and *ANKDRD11*) have poorer outcomes [10], [23]. Other undetected variants (i.e. another genetic disorder) should also be considered for atypical or unusual presentations of monogenic disorders, for example a previous child with KBG syndrome, epilepsy and developmental regression with *ANKRD11* mutation (Gly2006Argfs*26) was also diagnosed with cerebral folate deficiency [23]. While whole genome sequencing was not performed in our patient, dual pathology is unlikely as recent trio exome analysis was performed in 2021 and re-analysis in 2023 was also performed with no other candidate genes of interest arising (or metabolic markers detected in blood, urine, or CSF) to account for the severe epileptic dyskinetic encephalopathy phenotype. It is important to be aware that some phenotypes in KBG syndrome can be very severe, and complicated by developmental regression, often related to onset of severe epilepsy phenotypes, described in several patients [10], [23].

## Conclusion

In conclusion, genetic re-analysis of disorders of previous unknown aetiology is important for counselling, treatment, and understanding the wider phenotype and atypical presentations of gene variants in previously described classic syndromes such as KBG. Here we report a previously undescribed phenotype of *ANKRD11*-related KBG syndrome identified by trio exome sequencing (with previous negative 127 epilepsy gene panel and extensive metabolic investigation), characterised by a severe mixed hyperkinetic movement disorder in combination with early onset DEE. *ANKRD11* should be added to the list of causative genes linked to the umbrella term epileptic dyskinetic encephalopathy.

## Originality

The named authors confirm that this work is entirely original. Work of other authors has been sufficiently referenced when discussed in this article.

## Data access and retention

The principal author takes full responsibility for the data, the conduct of the research and the right to publish data separate and apart from any sponsor.

## Submission declaration and verification

This work has not been published before or currently under consideration for publication anywhere else.

## Informed consent

The named authors above confirm that the patient and their family have given verbal and written informed consent for this case report (with Video) to be written, presented, and submitted to a peer reviewed journal. This confidential consent form will be provided along with submission of the article for review.

## Declaration of artificial intelligence (AI) technologies

Eoin P. Donnellan, Nicholas M. Allen, Amre Shahwan or Kathleen Gorman have nothing to disclose regarding the use of Generative AI or AI-assisted technologies in the writing of this manuscript.

## Study funding

The authors report no targeted funding.

## CRediT authorship contribution statement

**Eoin P. Donnellan** and **Kathleen M. Gorman:** Writing – review & editing, Writing – original draft, Visualization, Validation, Supervision, Formal analysis, Conceptualization. **Amre Shahwan:** Writing – review & editing, Writing – original draft, Visualization, Validation, Methodology, Conceptualization. **Nicholas M. Allen:** Writing – review & editing, Writing – original draft, Visualization, Validation, Supervision, Software, Methodology, Investigation, Formal analysis, Data curation, Conceptualization.

## Declaration of competing interest

The authors declare that they have no known competing financial interests or personal relationships that could have appeared to influence the work reported in this paper.
