# Supplementary material for: Epileptic dyskinetic encephalopathy in KBG syndrome: Expansion of the phenotype
Source: Epilepsy Behav Rep. 2024 Jan 18;25:100647. doi: 10.1016/j.ebr.2024.100647 (PMC10839861; doi:10.1016/j.ebr.2024.100647)
Supplement: Supplementary data 3 [file mmc3.docx]

**Supplemental Data: Molecular diagnostics pipeline utilized in the diagnosis of the patient**

**SHORT VERSION**

Sequencing libraries were prepared for each sample from 50ng DNA using the Twist enrichment workflow (Twist Bioscience, San Francisco, CA) and a custom-design enrichment probe set (CeGaT ExomeXtra V3). Library preparation and capture was performed according to the manufacturer's instructions and paired-end sequencing was performed on a HiSeq/NovaSeq instrument (Illumina, San Diego, CA) with 2x100 base pairs (bp) read length. Sequence data were processed with Illumina bcl2fastq2. Adapter sequences were removed with Skewer and the sequences obtained, were aligned to the human reference genome (hg19) with the Burrows Wheeler aligner (BWA mem). Sequences that could not be clearly assigned to a genomic position were removed, as were sequence duplicates that were probably due to amplification (internal software). Sequence variants (single nucleotide exchanges and short insertions/deletions) were determined from the remaining high-quality sequences (CeGaT StrataCall).

Copy number variations (CNV) were computed on uniquely mapping, non-duplicate, high quality reads using an internally developed method based on sequencing coverage depth. Briefly, we used reference samples to create a model of the expected coverage that represents wet-lab biases as well as intersample variation. CNV calling was performed by computing the sample's normalized coverage profile and its deviation from the expected coverage. Genomic regions were called as variant if they deviate significantly from the expected coverage.

Resulting variants were annotated with population frequencies from gnomAD (2.1/3.1) and an internal database (CeGaT), factoring in external databases (e.g. HGMD, ClinVar), and with transcript information from Ensembl, RefSeq, Gencode, and CCDS. All variants were manually assessed before inclusion in the final report, classified and reported based on ACMG/ACGS-2020v4.01 guidelines.

**DETAILED VERSION**

**Sequencing:** The coding and flanking intronic regions were enriched using in solution hybridization technology and were sequenced using the Illumina HiSeq/NovaSeq system.

**NGS based CNV-Calling:** Copy number variations (CNV) were computed on uniquely mapping, non-duplicate, high-quality reads using an internally developed method based on sequencing coverage depth. Briefly, we used reference samples to create a model of the expected coverage that represents wet-lab biases as well as inter-sample variation. CNV calling was performed by computing the sample's normalized coverage profile and its deviation from the expected coverage. Genomic regions are called as variant if they deviate significantly from the expected coverage.

Please note that next generation sequencing based detection of copy number variations has lower sensitivity/specificity than a direct quantification method, e.g. MLPA. The absence of reported CNVs therefore does not ultimately guarantee the absence of CNVs.

**Computational Analysis:** Illumina bcl2fastq2 was used to demultiplex sequencing reads. Adapter removal was performed with Skewer. The trimmed reads were mapped to the human reference genome (hg19) using the Burrows Wheeler Aligner. Reads mapping to more than one location with identical mapping score were discarded. Read duplicates that likely result from PCR amplification were removed. The remaining high-quality sequences were used to determine sequence variants (single nucleotide changes and small insertions/deletions). The variants were annotated based on several internal as well as external databases.

**Diagnostic data analysis:** Variants were classified based on ACMG guidelines (Richards at al., 2015, PMID: 25741868).

Only variants (SNVs/Small Indels) in the coding region and the flanking intronic regions (±8 bp) with a minor allele frequency (MAF) < 1% are evaluated. Known disease-causing variants (according to HGMD) are evaluated in up to ±30 bp of flanking regions and up to 5% MAF. Minor allele frequencies are taken from public databases (e.g. gnomAD) and an in-house database. X-chromosomal variants that are listed in public databases equal to or greater than 50 times in a hemizygous state and are not disease-causing variants according to HGMD are excluded from analysis. If an acceptable sequencing-depth per base is not achieved by high-throughput sequencing, our quality guidelines demand local re-sequencing using classical Sanger-technology. Candidate CNV calls are evaluated manually. Potentially pathogenic findings are validated with a second method, like MLPA, on a case by case basis.

**Trio analysis:** Variants found in the patient and in the patient's parents were compared and filtered for four cases: *de novo* in the patient, patient is compound heterozygous, patient is homozygous and the parents are heterozygous, patient is hemizygous and the mother is heterozygous for variants on the X-chromosome.

Variants identified through exome analysis were evaluated with reference to the indicated phenotype. Therefore, single heterozygous variants in genes associated with autosomal recessive inheritance may not have been reported.

97.88%, 97.79%, and 97.89% of the targeted regions were covered by a minimum of 30 high-quality sequencing reads per base for the index, mother, and father, respectively.

**The evaluation of variants is dependent on available clinical information at the time of analysis.** The medical report contains all variants not classified as benign or likely benign according to current literature. *In silico* prediction of variants listed in the chart above is calculated on the basis of the output of the programs Mutation Taster, fathmm, Mutation Assessor, SIFT, fathmm-MKL coding, LRT, and PROVEAN according to the following criteria: 100% consensus = pathogenic/benign, ≥ 75% consensus = mostly pathogenic/benign, consensus < 75% or no prediction possible = inconsistent. SpliceAI was utilized to evaluate the consequence of variants on splicing (thresholds: 0.8-1 "splice effect", 0.6-0.8 "possible splice effect", <0.6 "no splice effect"; Jaganathan et al., 2019, PMID: 30661751). The prediction of a splice effect for missense variants is only indicated if a threshold of 0.8 is reached. This prediction can be complemented with additional *in silico* predictions in individual cases.

**Acknowledgements:** We would like to thank Dr. Kathrin Rothfelder (Center for Human Genetics, Tuebingen) for analyzing the NGS data generated by CeGaT Diagnostics and identifying the clinically relevant variant.
